# Development and validation of the Ibadan Simplified Developmental Screening chart

**DOI:** 10.3389/fped.2022.1055997

**Published:** 2023-01-11

**Authors:** I. A. Lagunju, Y. Adeniyi, A. E. Orimadegun, D. Fernandez-Reyes

**Affiliations:** ^1^ Department of Paediatrics, College of Medicine, University of Ibadan and Department of Paediatrics, University College Hospital, Ibadan, Ibadan, Nigeria; ^2^ Department of Psychiatry, College of Medicine, University of Ibadan and Department of Child and Adolescent Psychiatry, University College Hospital, Ibadan, Nigeria; ^3^ Department of Paediatrics, Institute of Child Health, College of Medicine, University of Ibadan, Ibadan, Nigeria, University College Hospital, Ibadan, Nigeria

**Keywords:** development, screening tool, disability, childhood, Africa

## Abstract

**Background:**

Developmental assessment remains an integral part of the routine evaluation of the wellbeing of every child. Children in resource-poor countries are not routinely assessed for signs of developmental delay and developmental disorders are frequently overlooked. A major gap exists in the availability of culturally appropriate and cost-effective developmental screening tools in many low and middle income countries (LMICs) with large populations.

**Objective:**

To bridge the existing gap, we describe the process of the development and validation of the Ibadan Simplified Developmental Screening (ISDS) chart, for routine developmental screening in Nigerian children.

**Methods:**

We developed an item pool across 4 domains of development namely, the gross motor, vision-fine motor, communication and socio-behavioural domains. The ISDS chart consists of 3–4 item questions for each domain of development, and responses are to be provided by the caregiver. Each chart is age-specific, from 6 weeks to 12 months. A total score derived from the summation of the scores in each domain are plotted on the ISDS scoring guide with a pass or fail score. Each child was evaluated by the Ages and Stages Questionnaire as the standard.

**Results:**

A total of 950 infants; 453 males and 497 females were enrolled. The estimates of internal consistency between the two instruments ranged between 0.7–1.0. Using the ASQ as the gold standard, the ISDS chart demonstrated a sensitivity of 98.8%, 78.4% and 99.7% in the gross motor, communication and the social and emotional domains respectively, for detecting infants who might require further assessment for developmental delays.

**Conclusion:**

The indigenous tool fills a major gap in the need for cost-effective interventions for developmental monitoring in LMICs. Future work should include the deployment of the tool in the wider population, using digital health approaches that could underpin policy making in the region.

## Introduction

There is a huge burden of developmental disabilities in the developing countries (1). More than 200 million people in low and middle-income countries are estimated to have developmental disabilities, with the majority being diagnosed late and having poor outcomes (2, 3). Early identification and timely interventions have long been recognised as the most effective ways of ensuring the best outcomes for children with developmental disorders (4, 5).

Addressing neurodevelopmental disabilities has become more important as significant reductions in infant and child mortality have been recorded in recent years (6). The sustainable development goals (SDGs) provide a framework for policy and action to address the needs of children with or at risk of developmental disorders, especially in resource-poor countries. Unfortunately, many children with developmental disabilities in LMICs are often denied access to appropriate educational opportunities or the acquisition of skills to sustain employment in the future. Other far-reaching implications include lower health status in children, malnutrition, stigmatisation, and a heavy economic and psychosocial burden on the family (7, 8).

There is a disproportionately higher burden of developmental disabilities in the LMICs and it has been estimated that 95% of children with developmental disabilities live in these countries (9, 10). Early identification of infants at risk of neurodevelopmental disorders is a major prerequisite for intervention programmes which significantly affects outcome. It ensures that interventions that aim at positively modifying the natural history of these disorders can start in the first weeks or months of life (11, 12).

Africa is home to about 120 million children who are under the age of five years, and this number accounts for 20% of the world's under-five population (13). In Nigeria, the most populous country in Africa, there are few professionals to attend to the health needs, including monitoring of the developmental trajectories of this large population of children (14, 15). As a result, the few available resources and the few professionals are often overburdened and do not have sufficient time for an elaborate assessment. A simple-to-use screening instrument is therefore necessary to reach more children and to identify those with developmental delays and disorders to ensure early intervention. While current standardised tools from western developed countries have been adapted for use in LIMC populations, the transfer of western-based tests to the African context is associated with significant limitations in score interpretation, cultural appropriateness, and feasibility of use in resource-constrained settings (16).

We aimed to develop a set of test items across the domains of development to conceptualise and develop the ISDS Chart (stage I), test its content validity in a small pilot study (stage II), test the internal and concurrent validity of the ISDS in a larger cohort (stage III) and evaluate the test-retest reliability of the tool (stage IV). Our study therefore set out to bridge this gap by developing a novel, cost-effective, simple, and easy-to-administer developmental screening tool as an indigenous, culturally appropriate, and readily available tool, to facilitate prompt identification of infants and young children at risk of developmental disabilities in our setting.

## Methodology

### Ethical approval

The study was carried out in accordance with the Declaration of Helsinki declarations. Ethical approval for the study was given by the University of Ibadan/University College Hospital Ethical Review Committee (ID UI/EC/18/0143). After explaining the purpose of the study and that no harmful or invasive procedures would be used, written consent was obtained from each child's parents or guardians for participation in the research.

### Study design

**Figure 1:**
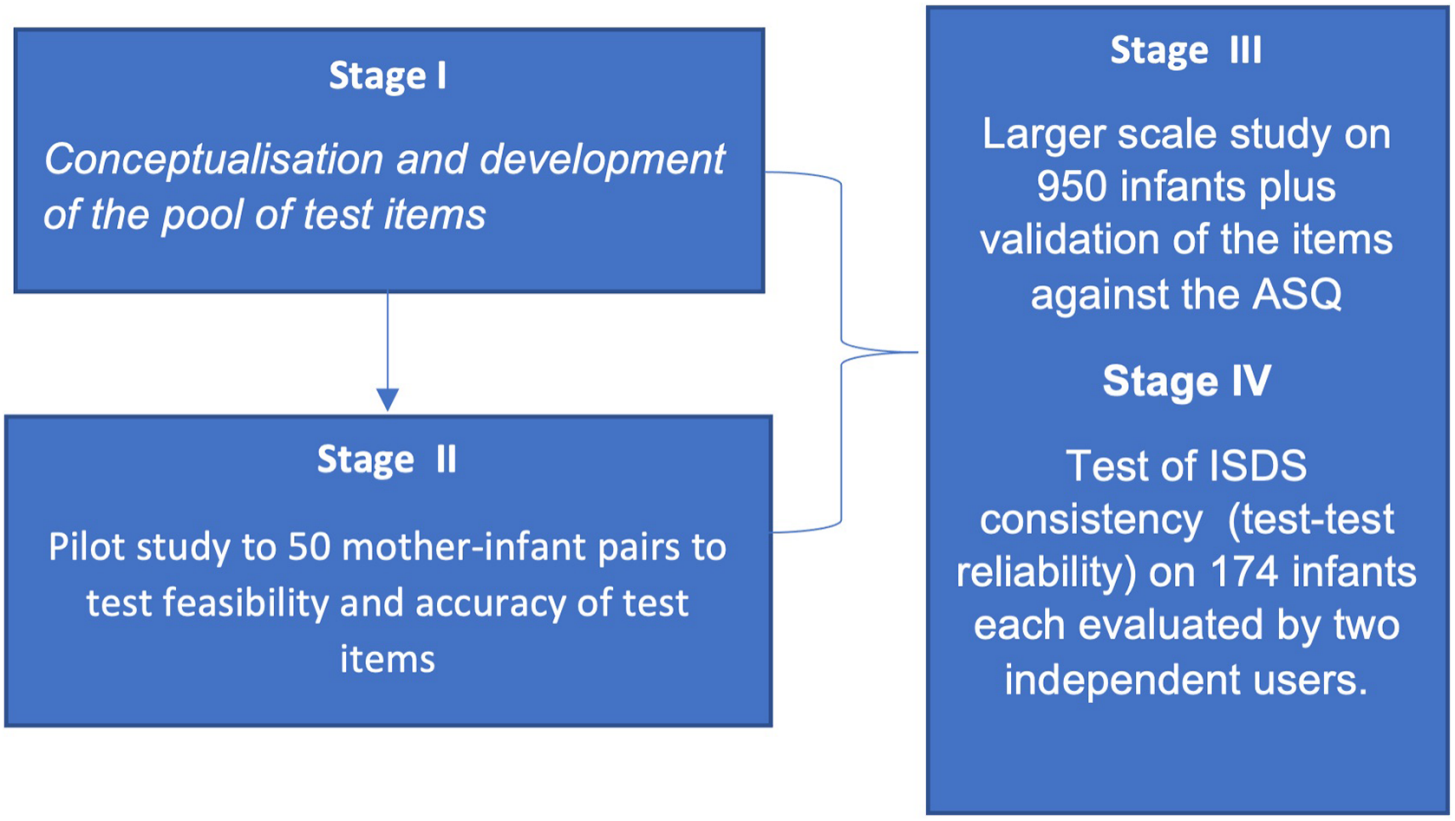
Flow chart for the development of screening tool.

This study was carried out in four stages (I–IV) as shown in Figure 1. Stage I focused on the conceptualisation and development of the novel ISDS Chart. This stage involved the development of lists of questions that constitute the items that make up the novel “Ibadan Simplified Developmental Screening” (ISDS) instrument for each age category and the review by two experts to establish the content validity. Stage II was a test of the context validity of the ISDS Chart. This was a pilot study on 50 infant-mother pairs to test its feasibility in the context of the target population and to test the usability (ease of use and understanding) of the ISDS chart.

Stage III of the work aimed at testing the internal and concurrent validity of the ISDS Chart. This was a larger study on 950 infants to investigate the internal and concurrent validity of the ISDS chart using the ASQ-3 as the gold standard. The sample size was derived by assuming a minimum acceptable sensitivity and sensitivity of 70%, and that one out of every ten children screened with the newly-developed ISDS Chart has developmental delay and requires expert evaluation, 807 participants will be needed to achieve a precision of 0.1 at a confidence level of 95%. Buderer's sample size formula for sensitivity and specificity was used to calculate this estimated sample size. Adjusting for a 15% non-response rate, the required sample size increased to 950 participants (17).

Stage IV was undertaken to test the internal consistency of the ISDS Chart. Two non-experts administered the ISDS to 174 infants to evaluate its test-retest reliability.

### Stage I: conceptualisation and development of the novel ISDS

The complete ISDS kit consists of the ISDS Chart (Supplementary File S1), which contains the test items for each specific age; the scoring guide for the specific age; and a set of simple toys and everyday items that are locally available and readily familiar to the child, which are used for evaluation of the infants in addition to the responses provided by the caregiver/mother. The ISDS Chart has a section for the documentation of the basic demographic information on the child, and this includes the name, date of birth, date of evaluation, age at evaluation, and the gestational age at delivery to determine if the child was term or preterm at delivery. There is also a provision for the documentation of any concerns that the caregiver might have with the child's development and functioning.

The ISDS items were created in four developmental domains: gross motor, vision, and fine motor; communication (hearing, speech, and language); and social/behavioural (social, emotional, and behavioural domains). We conducted a thorough review of the literature regarding normal ages for attaining the major developmental milestones for each domain of development in children from various cultures. We drew on experts' knowledge and experience, as well as available literature, to identify the significant developmental milestones in each domain at the precise age targets (18–21). The questions were then written in a way that eliminated ambiguity in order to be easily understood by the caregivers. After that, experts reviewed the ISDS charts and scoring guide before the pilot study.

The proposed ages of screening were synchronised with the ages at which routine vaccinations are delivered to infants in Nigeria under the National Programme for Immunisation (NPI) Schedule. Thus, the ISDS items were created for ages 6 weeks, 10 weeks, 14 weeks, 6 months, 9 months, and 12 months (22).

### Stage ii: context validity of the novel ISDS

During stage II of the study, a pilot study on 50 mother-infant pairs was undertaken to test the feasibility of the use of the ISDS Chart. The tests were undertaken in the routine infant immunisation clinic of the University College Hospital, Ibadan, Nigeria. The test items were fine-tuned based on the experience with the administration of the screening chart in the pilot study.

### Stage III: internal and concurrent validity of the novel ISDS

In a cross-sectional study involving 950 infants, which utilised the ISDS Chart as the experimental screening tool and the Ages and Stages Questionnaire-3 (ASQ-3) as the gold standard for the detection of developmental delay in infants aged 6 weeks to 12 months, the participants were continence samples of infants using a non-probability sampling method, who presented at the Immunisation Clinic of the Adeoyo Maternity Hospital, Ibadan, Nigeria, a secondary health facility; and the Immunisation Clinic of the University College Hospital, Ibadan, Nigeria, a tertiary health facility.

The Ages and Stages Questionnaire (ASQ-3) (23, 24) is applicable as a researcher-administered and self-administered assessment form. It is composed of 21 sets of questionnaires covering the age range of 2 to 60 months. The questionnaire covers the five key developmental areas, namely, gross motor skills, fine motor skills, communication skills, problem-solving/cognition skills, and social/personal interaction. Each set is composed of 30 items, 6 in each domain. Responses to items in all the domains are scored as follows: “yes” response (10 points), “sometimes” response (5 points) and “not yet” response (0 points). The maximum score in each domain is 60 points. Scores obtained from each domain are compared with established cut-off points at one and two standard deviations, which are used to identify children at risk of developmental delay. Referral for further assessment is advised if the score in any domain falls below the 2SD cut-off. If the score in any domain is within the one standard deviation (1SD) and two standard deviation (2SD) cut-off points, it is advised to provide learning activities and monitor the child's development. The ASQ has been proven to be reliable in detecting developmental delays in under-fives. A study reported adjusted sensitivity and specificity (95% confidence intervals) of 87.4% (62.9–96.6%) and 82.3% (80.5–83.9%), respectively (25). The ASQ has been used in the assessment of children in many low and middle-income countries (26).

Two study sites were utilised: the Immunisation Clinic of the Adeoyo Maternity Hospital, Ibadan, Nigeria, a secondary health facility; and the Immunisation Clinic of the University College Hospital, Ibadan, Nigeria, a tertiary health facility. The performance of each child on the ISDS Chart and the ASQ-3 was documented.

### Stage iv: test-retest reliability of the ISDS chart

This stage was undertaken to evaluate the internal consistency of the ISDS Chart. Two non-experts administered the ISDS Chart on each child, at the same site and setting, on the same day. A total of 174 infants participated in this stage to determine the test -retest reliability of the tool.

### Data analysis

All data were entered and analysed using the Stata BE. 17.0 for Windows (Stata Corp LLC, USA). The sum of the scores for each domain by age groups were determined and the distribution of the ASQ-3 and ISDS scores was examined by measuring the median, mean, and standard deviations (SD) for the study participants' age groups. We also used the Wilcoxon signed-rank test to compare the ASQ-3 development ages to those of the ISDS since the data are non-parametric in nature. Cronbach's alpha was used to measure the internal consistency of each subscale of the ISDS charts. Spearman's rank correlation coefficient between the ASQ-3 score and the ISDS scores were calculated for each developmental area. According to Tavakol and Dennick (27), a correlation greater than 0.60 suggests a high level of internal consistency. The validity of the ISDS in detecting children that would require further developmental assessment was assessed by calculating the sensitivity and specificity, using the ASQ-3 as the gold standard. The optimal cut-off points of ISDS scores of each domain for detection of developmental delayed identified by ASQ-3 was determined using the CUTPT Stata module for empirical estimation of cut-off point for a diagnostic test. The area under the ROC curve (AUC) was used to summarize the overall diagnostic accuracy of the ISDS as a tool for the assessment of development among Nigerian infants. An AUC of 0.5 suggests no discrimination, ability to detect a child that would fail on the ASQ-3 scale, 0.7 to 0.8 was considered acceptable, 0.8 to 0.9 was considered excellent, and more than 0.9 is considered outstanding (28). To assess the reliability of ISDS chart as a screening tool for identifying children who would benefit in further evaluation, two non-experts administered the ISDS to 174 infants and correlations between the test two test scores were determined.

## Results

### Conceptualisation and development of the novel ISDS

The test items were designed to be simple questions that can be easily understood by healthcare workers at the primary level of care as well as caregivers/mothers of any level of education. The goal of each item question is to evaluate whether the child does or is able to perform the activity in the test item. Each domain has 3 or 4 questions, with a total of 12–16 items of questions for each age-specific chart. The ISDS Chart is to be completed by the healthcare worker with the responses provided by the mother or caregiver of the infant. The responses of the caregiver were corroborated by direct observations of the healthcare worker who administered the ISDS screening test. The ISDS screening test requires that every single test item be evaluated. It was designed as a researcher-administered tool but could also be used as a self-administered tool by mothers or caregivers.

### Scoring of test items

The scoring of the test item is on a scale of 0–5 and the caregiver is required to provide one of three responses to each given item of testing; “yes” if the child performs the task/activity well and comfortably; “no” if the child is not yet able to and does not perform the activity; and “somewhat” if the caregiver has observed the activity in the child but not consistently. The responses “yes”, “somewhat” and “no” are scored as 5, 2, and 0 points, respectively. The screening test requires that every single activity on the ISDS Chart for the age group be evaluated and scored based on the guide provided. At the end of the evaluation, the total score for each domain of development is determined and plotted on the ISDS Chart scoring guide. The scoring guide provides specific cut-off points for each domain of development tested. The scoring guides are specific for each age of testing. The score is thereafter plotted in the appropriate box, coloured red and white. The scoring categorised each child into one of two groups; a score in the red box is an indication for referral and further evaluation, while a score in the white box indicates that the child does not require any further evaluation at the point in time (Supplementary File S2).

## Internal and concurrent validity of the novel ISDS

### Characteristics of the study participants

A total 950 infants participated in the Stage III of the study comprising 453 (47.7%) males and 497 (52.3%) females. The age of participants ranged from 6 weeks to 12 months, and they were categorised into six age groups, namely, 6 weeks (*n* = 206; 21.7%), 10 weeks (*n* = 175; 18.4%), 14 weeks (*n* = 185; 19.5%), 6 months (*n* = 108; 11.4%), 9 months (*n* = 198; 20.8%) and 12 months (*n* = 78; 8.2%). Other descriptive characteristics of the children enrolled into the study are as shown in Table 1. The sex distribution of the participants was not statistically different, with the exception of children who had a background history of neonatal jaundice, which was significantly higher in the male (62.0%) group than the female (38.0%) group. Twelve (1.3%) of the mothers expressed concerns about their child's development, including concerns about vision (*n* = 4; 0.4%) and behaviour (*n* = 8; 0.9%).

**Table 1:** Distribution of study Participants’ characteristics by sex.

	Total	Male		Female		*p*
		*n*	%	*n*	%	
All participants	950	453	47.7	497	52.3	–
**Age**						
4–8 weeks	206	103	50.0	103	50.0	0.326
9–12 weeks	175	84	48.0	91	52.0	
13–16 weeks	185	86	46.5	99	53.5	
5–7 months	108	60	55.6	48	44.4	
8–10 months	198	89	45.0	109	55.0	
11–14 months	78	31	39.7	47	69.3	
**Gestational age**						
Preterm	71	32	45.1	39	54.9	0.647
Term	879	421	47.9	458	51.1	
**Neonatal jaundice**						
Yes	50	31	62.0	19	38.0	0.037
No	900	421	46.8	479	53.2	
**Perinatal asphyxia**						
Yes	9	5	55.6	4	44.4	0.744*
No	941	447	47.5	494	52.5	
**Neonatal seizure**						
Yes	4	1	25.0	3	75.0	0.626
No	946	451	47.7	495	52.3	

* Fisher's exact test.

### Assessment of the ISDS internal consistency

The ISDS consists of four subscales which assess the “vision and fine motor”, “hearing, speech, and language (communication)”, “gross motor”, as well as “social, emotional and behavioural” domains of development, respectively. For each domain of development, the number of test items, mean of the total scores and the estimates of the internal consistency (Cronbach alpha) are as shown in Table 2. The estimates of the internal consistency (Cronbach alpha) fell within the acceptable range of 0.7 to 1.0 except in the gross motor domain among participants assessed on the 10 weeks ISDS instrument which was 0.64. For the subscales of hearing, speech, and language (communication) and gross motor skills, the internal consistency reliability increased with age from 6 weeks to 12 months, while the internal consistency reliability demonstrated no consistent trend in the subscales of vision and fine motor and social and behavioural skills.

**Table 2:** Summary Statistics of Scores and Internal Consistency of the ISDS Scales by Domains and Ages.

ISDS domains	6 weeks	10 weeks	14 weeks	6 months	9 months	12 months
**Hearing and speech**						
No. of items	3	4	3	4	3	3
Min—max	5–15	10–20	10–15	5–20	10–15	0–15
Mean ± SD	14.8 ± 1.1	19.5 ± 1.7	10.6 ± 1.6	19.6 ± 1.8	14.0 ± 1.8	12.1 ± 4.1
Cronbach alpha	0.71	0.75	0.778	0.77	0.77	0.82
**Gross motor**						
No. of items	3	3	3	3	3	3
Min—max	5–15	5–15	2–15	5–15	0–15	0–15
Mean ± SD	14.8 ± 1.0	14.8 ± 1.0	14.1 ± 2.0	14.4 ± 1.6	14.7 ± 1.6	16.7 ± 1.8
Cronbach alpha	0.74	0.64	0.77	0.80	0.89	0.91
**Vision and fine motor**						
No. of items	4	3	3	4	3	4
Min—max	12–20	7–15	5–15	0–20	5–15	0–15
Mean ± SD	19.7 ± 1.2	14.8 ± 0.9	14.4 ± 1.8	18.7 ± 2.8	14.4 ± 1.7	14.3 ± 2.2
Cronbach alpha	0.75	0.70	0.78	0.74	0.79	0.97
Social and behavioural						
No. of items	3	3	3	3	3	3
Min—max	5–15	5–15	10–15	5–15	0–15	5–15
Mean ± SD	12.9 ± 2.5	14.7 ± 1.2	12.2 ± 2.4	14.1 ± 2.1	13.1 ± 2.6	13.3 ± 3.2
Cronbach alpha	0.72	0.75	0.76	0.74	0.77	0.87

SD, standard deviation.

### Cut off and validity of ISDS compared with ASQ-3 scale

Table 3 presents the cut-off values for the ISDS scores, as well as their sensitivity and specificity for suggesting the need for further evaluation for developmental delay using the ASQ-3 as the gold standard. The ISDS chart demonstrated high sensitivity in the social and behavioural domains (99.7%), gross motor domain (98.8%), and hearing, speech, and language (communication) domains (78.4%). In the vision and fine motor domains (22.3%) and hearing and speech domains (82.6%), specificity ranged from moderate to high.

**Table 3:** Cut-off values for sum ISDS scores for the four domains of development.

Age and domains	Cut-off (cm)	Sensitivity	Specificity
**6 weeks**			
Hearing and speech	12.0	0.97	0.57
Gross motor	10.0	0.98	0.75
Vision and fine motor	12.0	0.99	0.51
Social and behavioural	10.0	0.77	0.59
**10 weeks**			
Hearing and speech	12.0	1.00	0.67
Gross motor	10.0	0.99	0.50
Vision and fine motor	10.0	0.99	0.80
Social and behavioural	12.0	0.95	0.30
**14 weeks**			
Hearing and speech	10.0	0.78	0.99
Gross motor	12.0	0.85	0.67
Vision and fine motor	12.0	0.94	0.81
Social and behavioural	10.0	0.80	0.84
**6 months**			
Hearing and speech	15.0	0.99	0.60
Gross motor	9.0	0.99	0.50
Vision and fine motor	15.0	0.84	0.50
Social and behavioural	12.0	0.82	0.24
**9 months**			
Hearing and speech	12.0	0.80	0.56
Gross motor	10.0	0.97	0.15
Vision and fine motor	10.0	0.91	0.71
Social and behavioural	10.0	0.73	0.44
**12 months**			
Hearing and speech	7.0	0.91	1.00
Gross motor	12.0	1.00	0.67
Vision and fine motor	15.0	0.93	1.00
Social and behavioural	12.0	0.77	0.40

**Figure 2:**
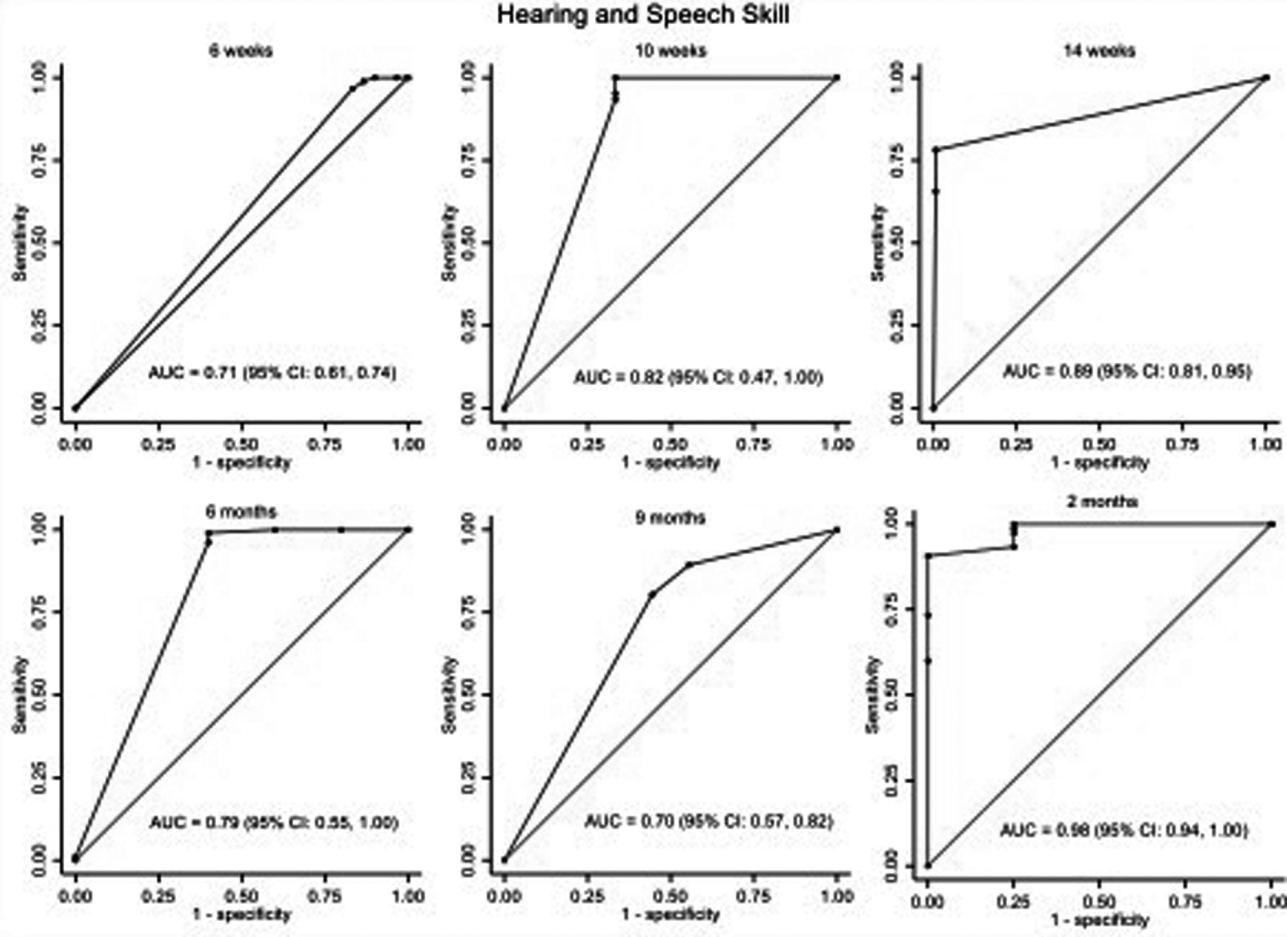
ROC curves showing the performance of ISDS in the detection of delay in hearing and speech domain at the age-specific optimal cut-off.

**Figure 3:**
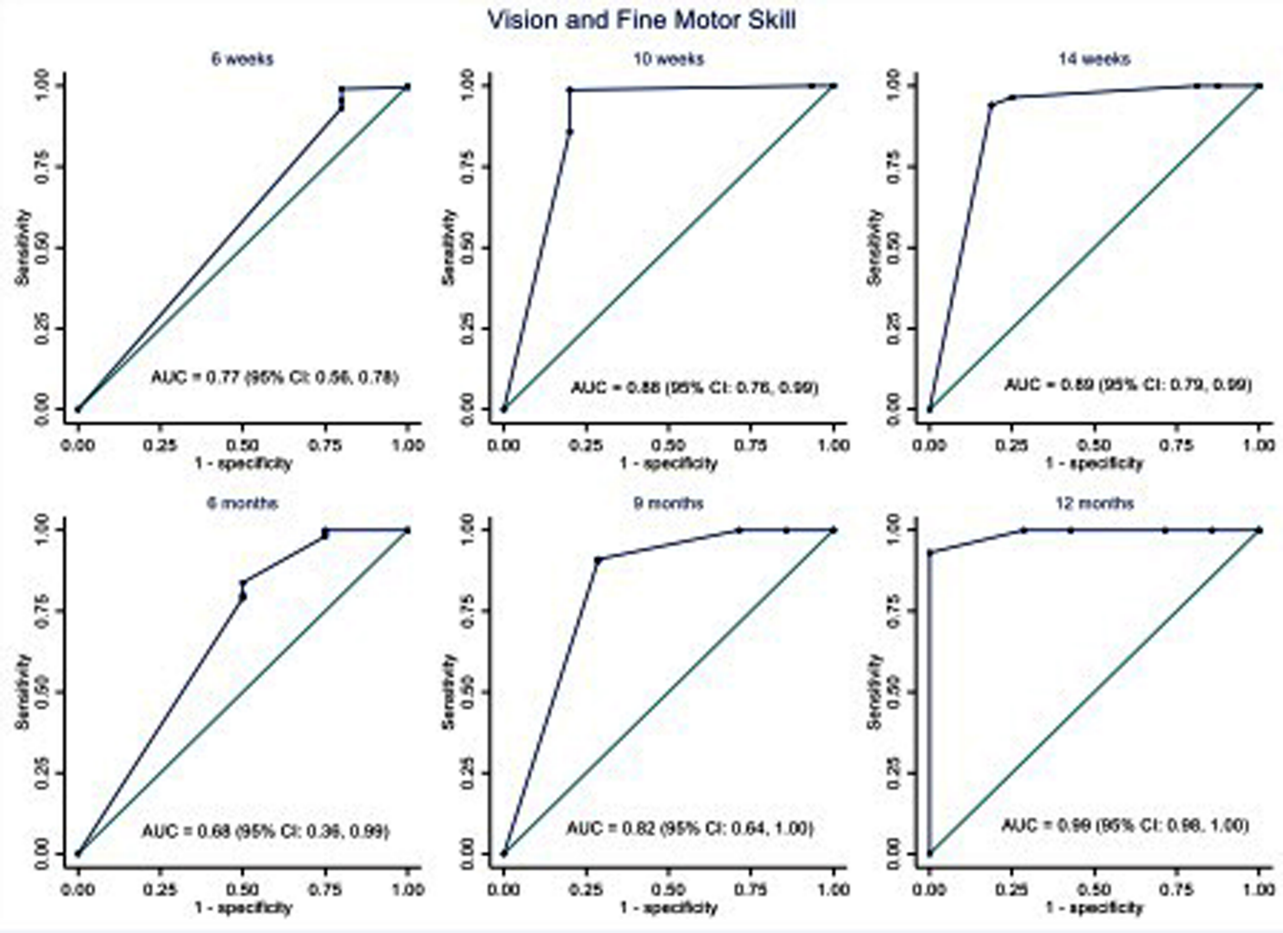
ROC curves showing the performance of ISDS in the detection of delay in vision and fine motor domain at the age-specific optimal cut-off.

**Figure 4:**
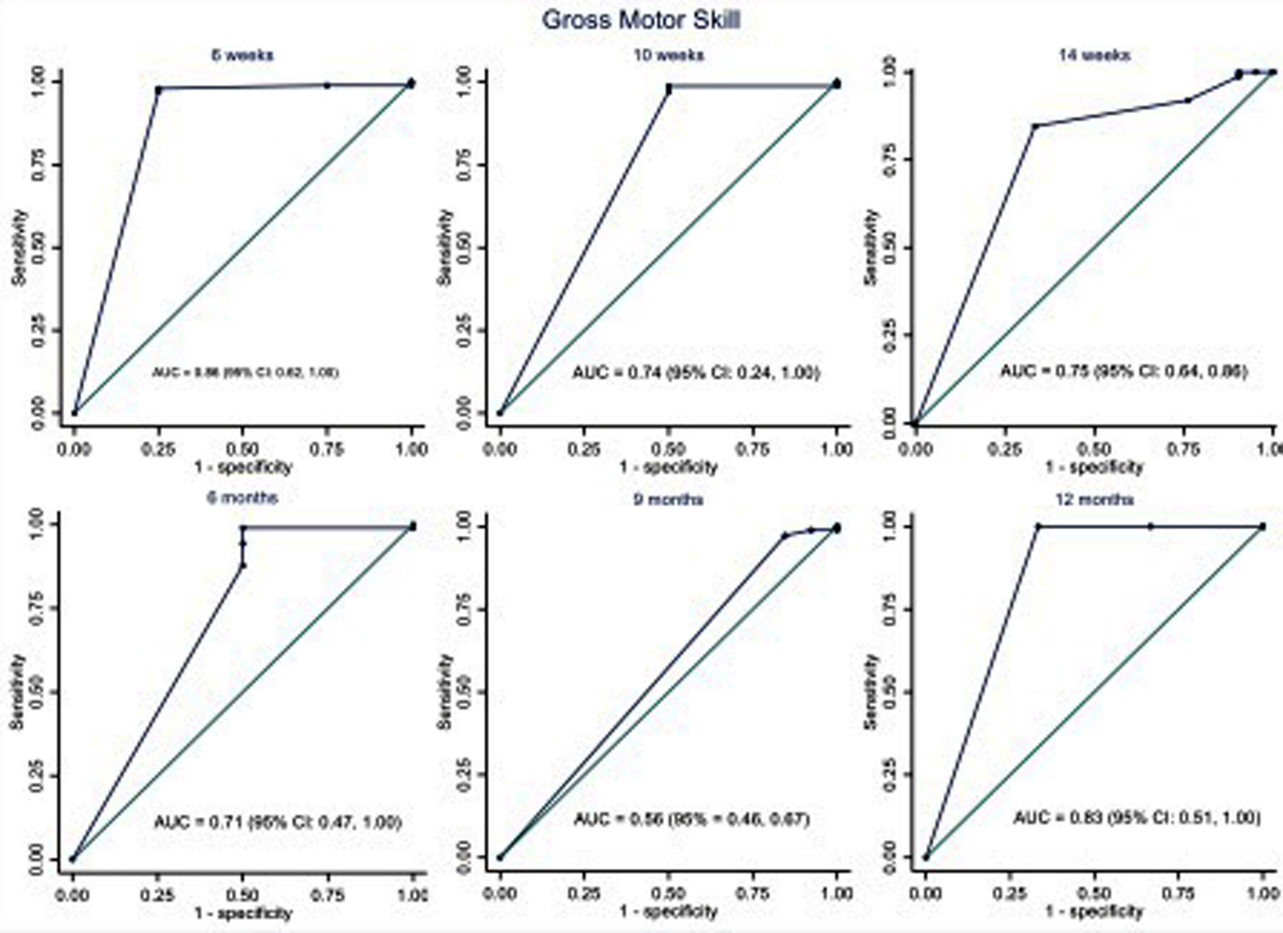
ROC curves showing the performance of ISDS in the detection of delay in gross motor domain at the age-specific optimal cut-off.

**Figure 5:**
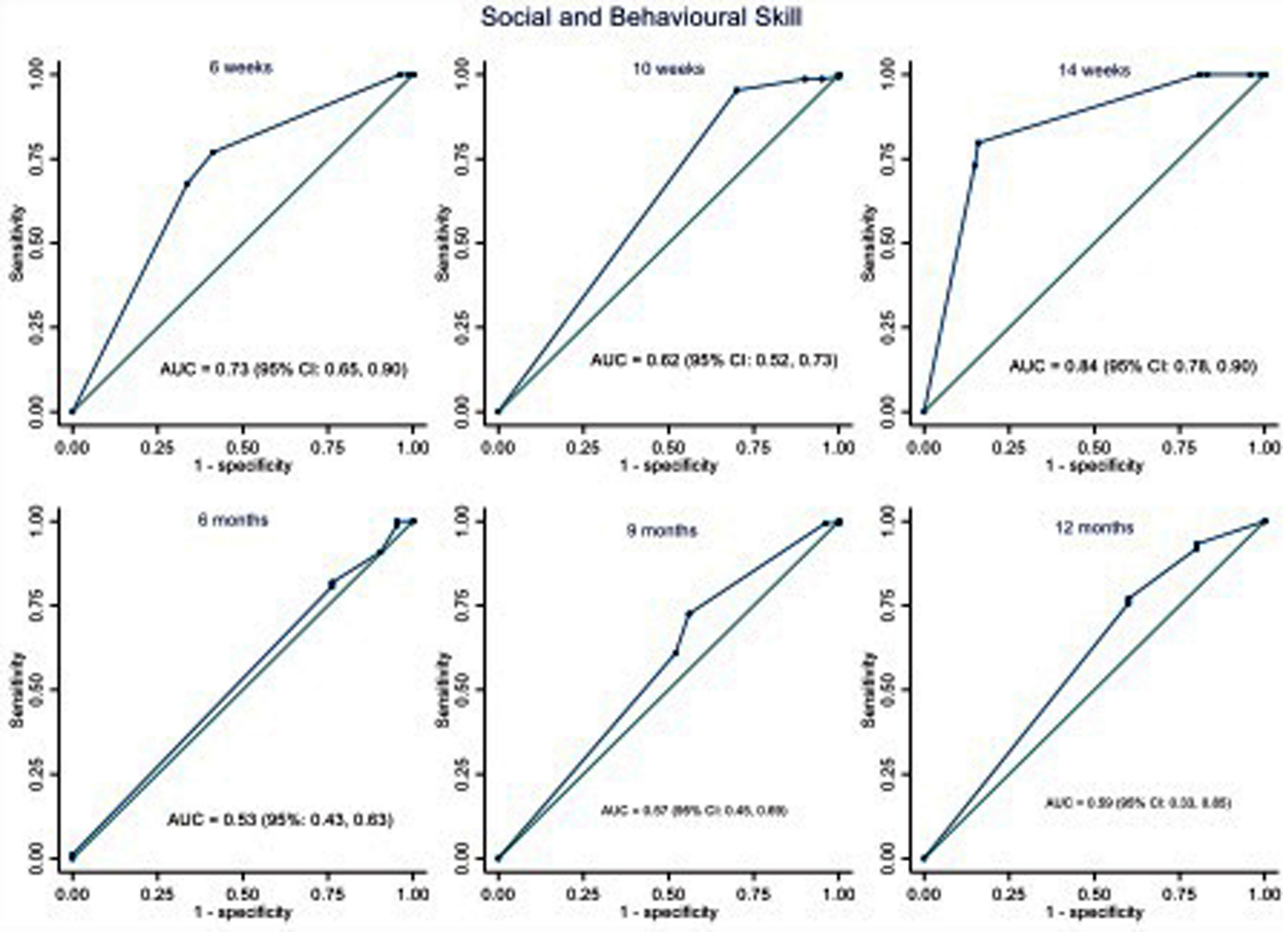
ROC curves showing the performance of ISDS in the detection of delay in social and behavioural domain at the age-specific optimal cut-off.

The area under the ROC curve (AUC) was used to assess the ISDS Chart's performance in detecting ASQ-3 failures, as illustrated in Figures 2–5. The ISDS chart has AUC values ranging from 0.71 (95% CI = 0.61, 0.74) at 6 weeks to 0.98 (95% CI = 0.94, 1.00) at 12 months of age, all of which are greater than the acceptable 0.7, as shown in Figure 2. Figure 3 shows that the ISDS chart exhibited AUC values above 0.7 in the vision and fine motor domains at ages 6 weeks, 10 weeks, 14 weeks, 9 months, and 12 months, but a slightly lower value at 6 months. Furthermore, for gross motor skills, the AUC values were above the acceptable 0.7 at all ages except 9 months, when it was 0.56 (95% CI = 0.46, 0.67) as shown in Figure 4. In comparison to other domains, the social and behavioural domain had an AUC value of 0.7 at 10 weeks, 6 months, 9 months, and 12 months, which were lower than acceptable (Figure 5).

### Test-retest reliability of the ISDS chart

**Figure 6:**
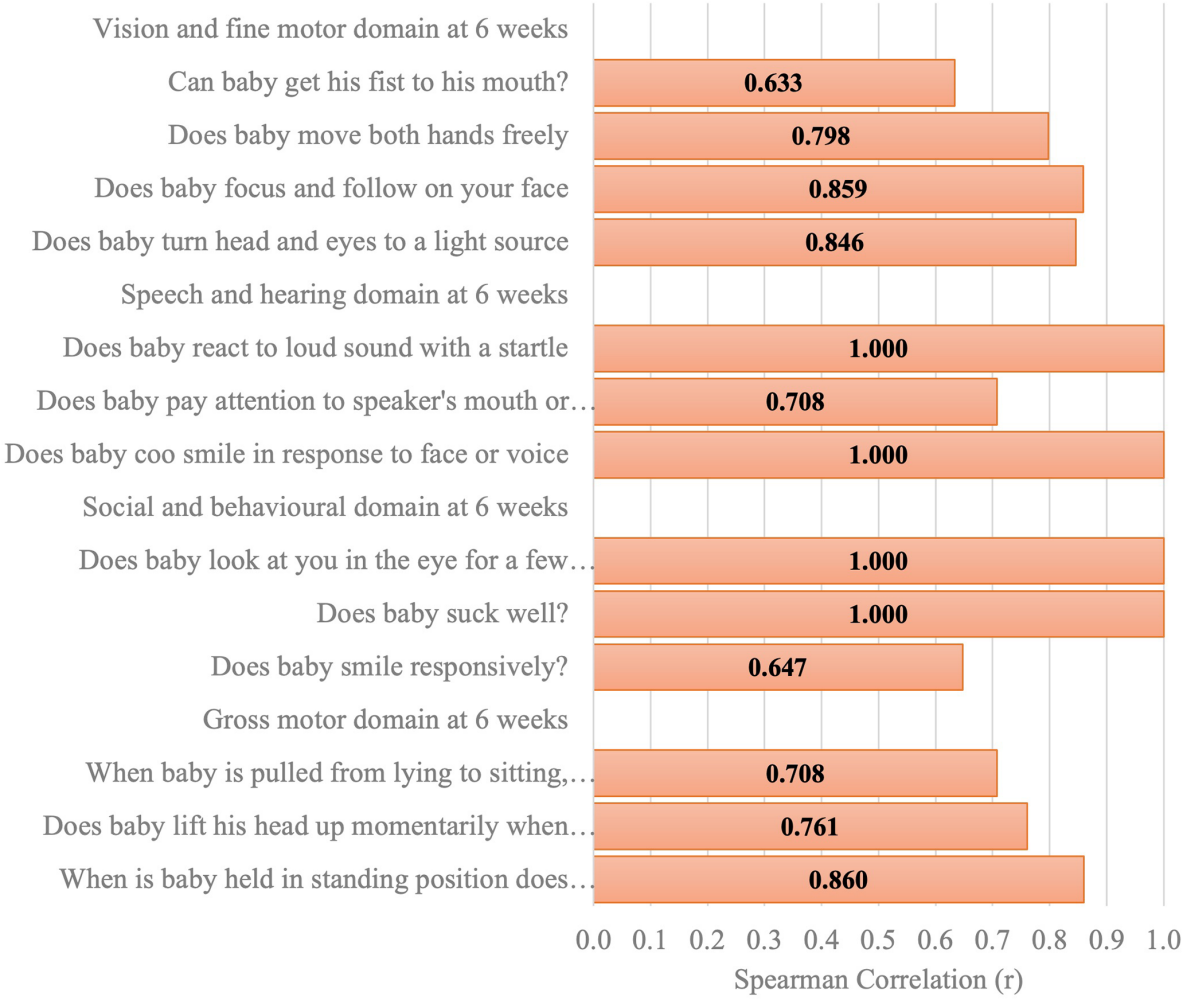
Spearman correlation coefficient of the ISDS scores of two assessors of the same participants aged 6 weeks.

**Figure 7:**
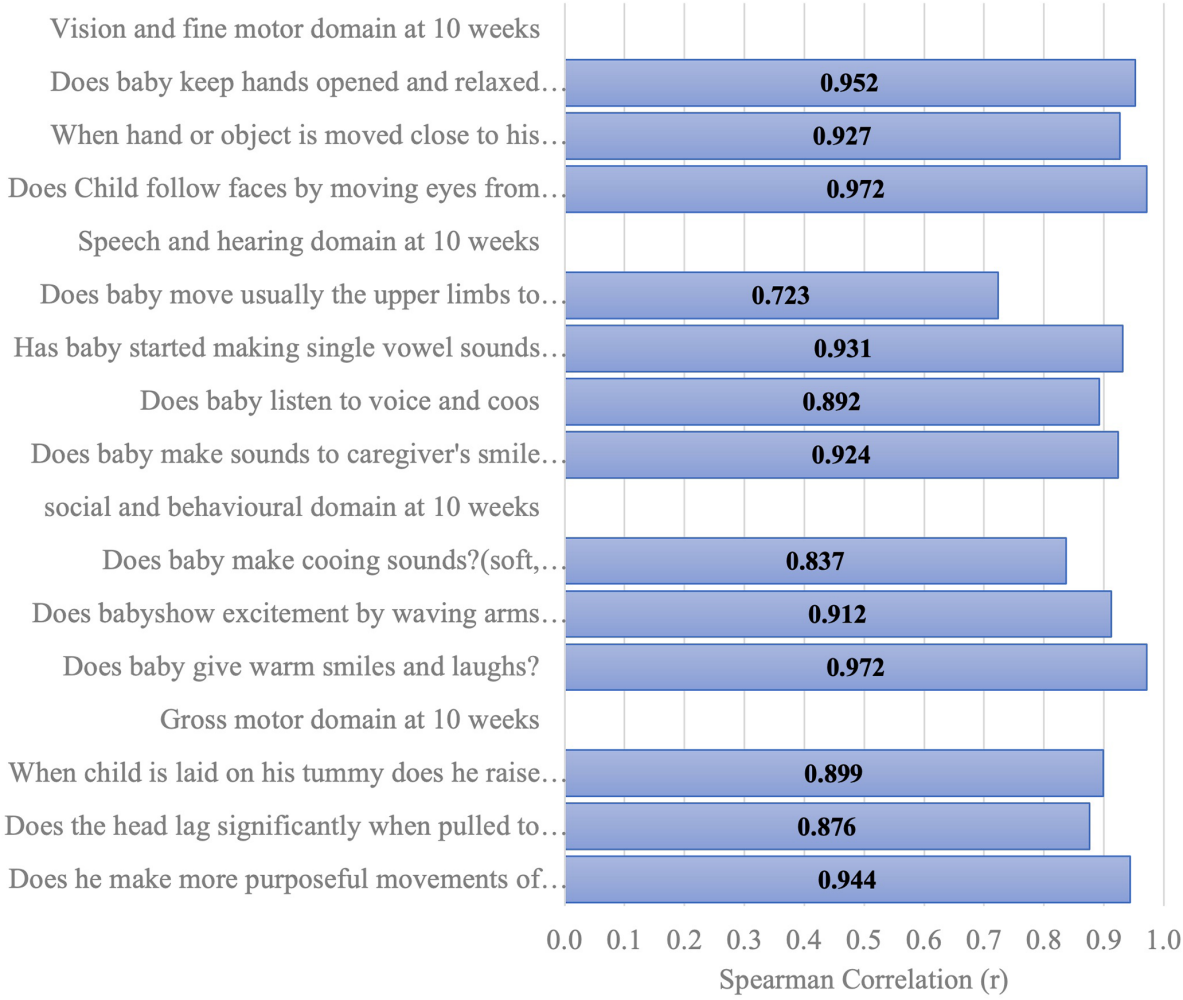
Spearman correlation coefficient of the ISDS scores of two assessors of the same participants aged 10 weeks.

**Figure 8:**
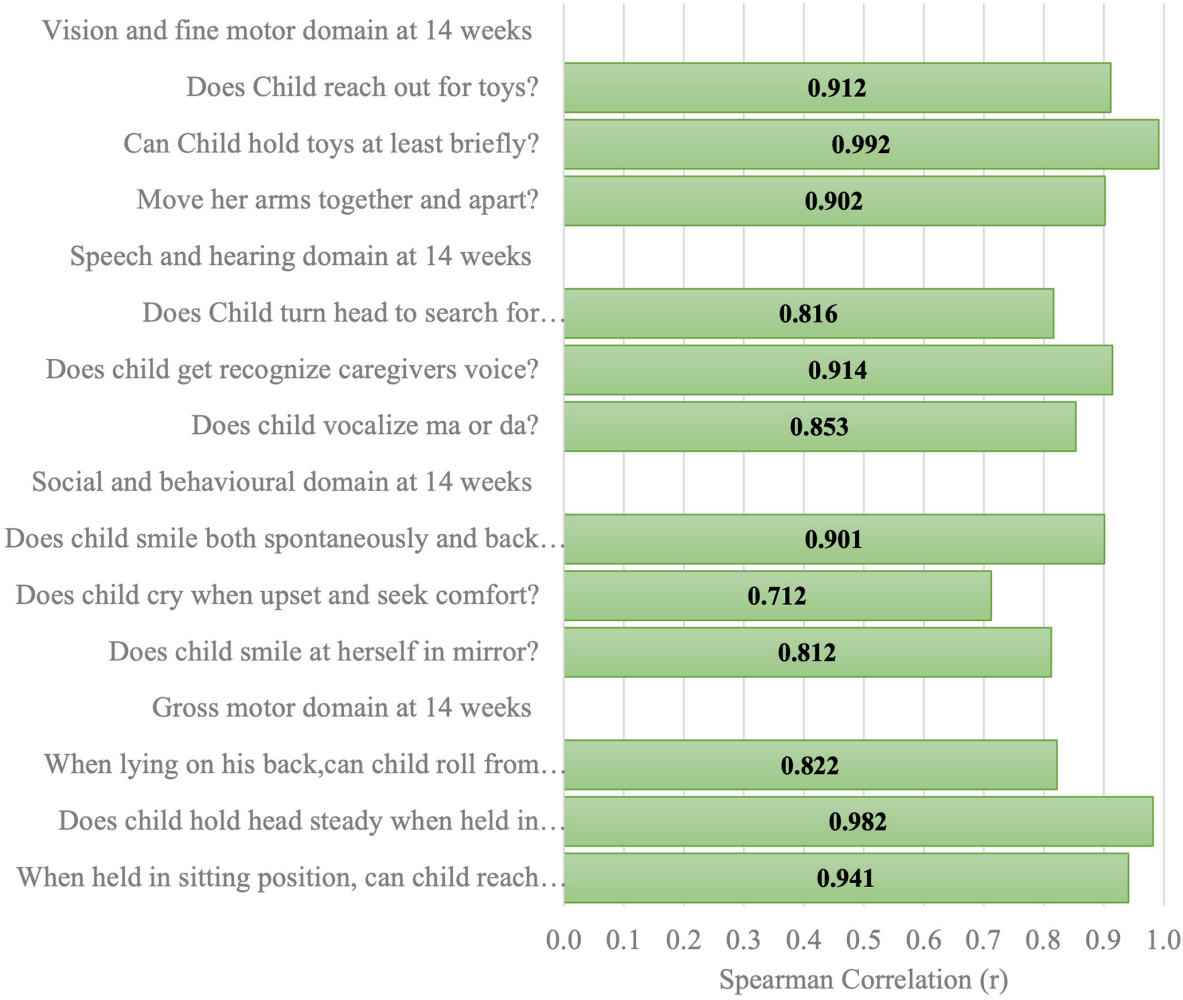
Spearman correlation coefficient of the ISDS scores of two assessors of the same participants aged 14 weeks.

**Figure 9:**
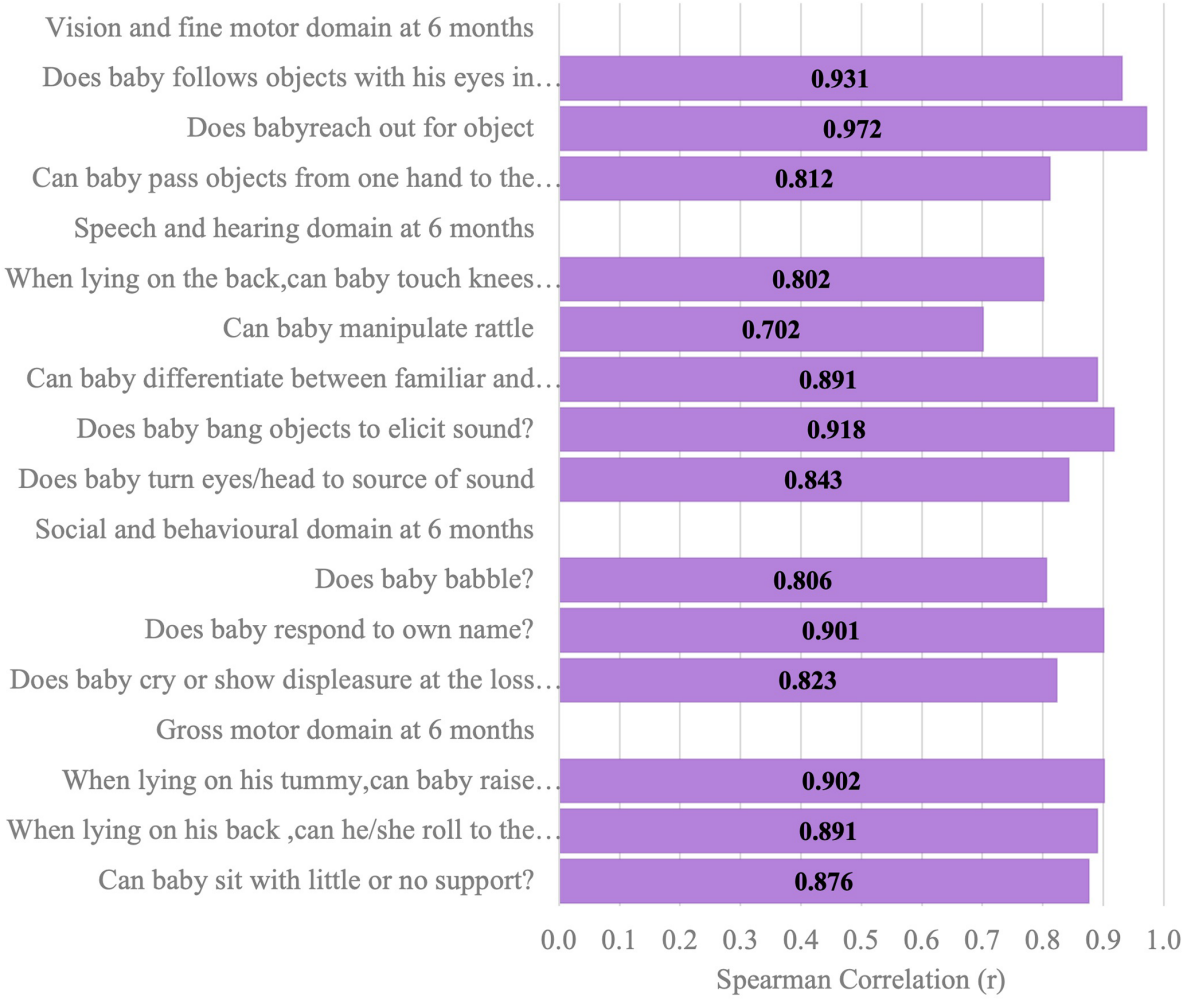
Spearman correlation coefficient of the ISDS scores of two assessors of the same participants aged 6 months.

**Figure 10:**
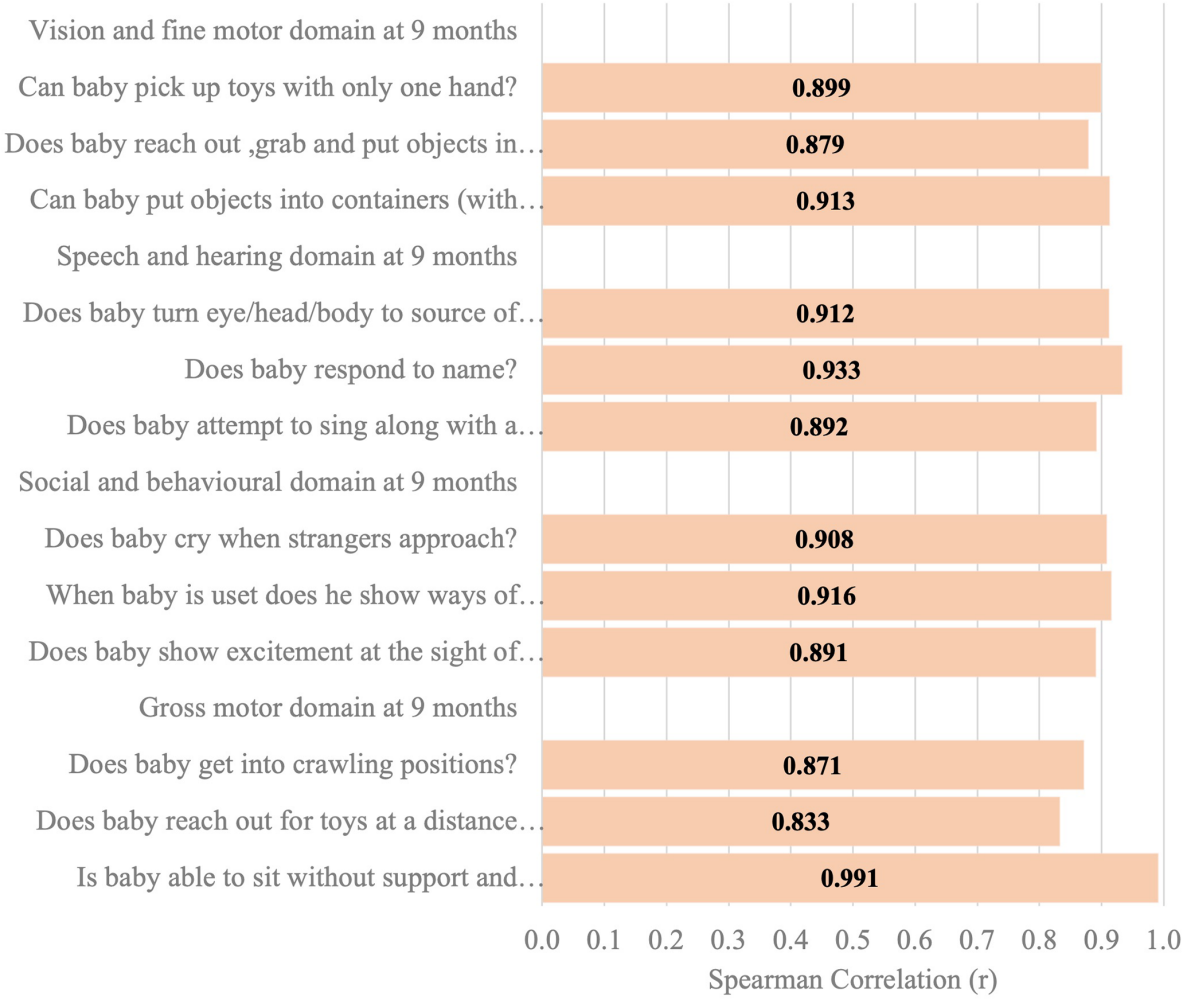
Spearman correlation coefficient of the ISDS scores of two assessors of the same participants aged 9 months.

**Figure 11:**
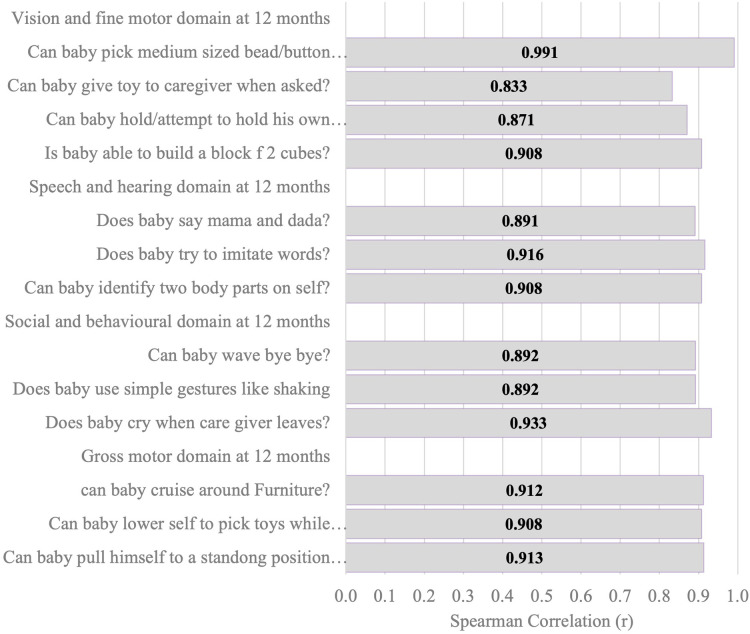
Spearman correlation coefficient of the ISDS scores of two assessors of the same participants aged 12 months.

Figures 6–11 show the Spearman rho coefficient of correlations between the scores acquired by two independent users of the ISDS chart on the same children in the same clinic on the same days. Notably, all of the items in each domain at all ages had significant correlation coefficient values, *r* > 0.7, *p* < 0.001 (Figures 6–11). The bars shown on Figures 6–11 present proof of high stability and degree of agreement between the two users' ISDS scores at 6 weeks, 10 weeks, 14 weeks, 6 months, 9 months, and 12 months, respectively.

## Discussion

In this study, we developed a novel, simple, and culturally sensitive screening instrument, the “Ibadan Simplified Developmental Screening” (ISDS) Chart, for screening children for developmental delays in infancy and also evaluated and demonstrated its validity and reliability for use in the most populous nation in Africa. The inclusion of early childhood development in the United Nations' Agenda for Sustainable Development raises questions about how this objective should be monitored, particularly in settings with limited resources. In addition, it emphasizes the importance of effective monitoring of developmental milestones and surveillance for deviations from the expected trajectory. Tracking child health and development in low-resource nations has been difficult due to a lack of appropriate developmental assessment tools and insufficient manpower to implement measurement (29).

As part of the efforts at developing tools for routine developmental assessments in children in LMICs, El Shafie and colleagues developed the Egyptian Developmental Screening Chart (EDSC) for children from birth up to 30 months (30). The EDSC has a set of checklists based on the Baroda Developmental Screening Test (BDST) questionnaire (31) which consists of 54 items carefully chosen from the 230 items in the Bayley's Scale of Infant Development. In the BDST, 22 items test the gross and fine motor functions while the remaining 32 items test of mental function representative of the cognitive, social and language domains. A *Z*-score chart for motor and mental development follow up was designed for each age group from birth to 30 months and a 97% pass level of development scores of children was taken as the reference. Any child's score below −2 SD was considered to be indicative of developmental delay and the need for further developmental monitoring. The EDSC was also compared with the ASQ and reported to have a sensitivity of 84.38% and a specificity of 98.36% for identifying Egyptian children who have delayed development.

The review by Neocleous and colleagues (32) on training packages for the use of child development tools in LMICs identified 24 tools used in LMICs but training information was available for only 18 tools. The study showed that the larger proportion of the tools were developed in the USA (6) while there were 2 from India and Bangladesh and one each from Cambodia, Malawi, Mexico and Mongolia. The study identified major gaps in the availability of training tools on the use of the developmental assessment tools used in LMICs. Tools developed in the Western countries used for developmental assessment in LMICs include the Denver's Developmental Screening test, PEDStest, Denver's Prescreening Developmental Questionnaire and the Infant Neurological International Battery (Infanib).

Current evidence therefore shows that many LMICs still assess child development using instruments or charts developed and validated in developed Western nations, such as the ASQ-3 (3, 33). Despite the fact that such established development charts are frequently translated into native African languages, these translations frequently disregard local customs and culture, leading to a misinterpretation of the results. Considering this, we designed, reviewed, and refined the ISDS chart, which evaluates the same set of domains as the ASQ, using our experiences and data from a large cohort of infants living in the densely populated city of Ibadan, Nigeria. The newly constructed ISDS chart was validated against the third version of the ASQ.

Overall, we found the ISDS chart to be a sensitive and reliable developmental delay screening tool for Nigerian infants. It is not time-consuming, no special testing equipment is required, and parents do not need to memorize developmental milestones. The design of the chart is straightforward and conceptually transparent, allowing child health professionals and parents to demonstrate a child's normal or delayed general development. It is useful for depicting a child's continued progress or lack thereof at follow-up. The ISDS Chart exhibited good to moderate and acceptable psychometric qualities as well as content, construct, and criterion validity. The ISDS's strong Cronbach alpha values in all domains at all ages revealed good and acceptable internal consistency, indicating evidence of its dependability. The ISDS Chart expands on the information and techniques of existing ASQ-3 development assessment tools, but it is designed to be used as a screening measure for clinical practice implementation in the context of the Nigerian new-born population. Our findings show that the ISDS, as a novel measure, could be effective in measuring development in Nigerian infants. Periodic developmental screening, it is believed, would result in prompt and correct identification of developmental deficits at each time point, thereby encouraging the need for further investigation and referral for specialist assessment by community workers and lower cadre healthcare providers.

Although the preliminary evidence of validity and inter-rater reliability need further confirmation in a larger multi-site study, the findings allow us to infer that the ISDS Chart satisfactorily identifies children at risk of developmental delay in our large-scale routine clinical practice. The next step in this research should be to figure out how to apply this novel, indigenous developmental screening tool in practice to raise disease awareness and promote healthy behaviour modification.

The ISDS Chart presents numerous benefits as a simple and novel tool for assessing children for developmental delays. It was developed utilising a comprehensive methodological approach that incorporated both qualitative and quantitative data, as well as best practises for assuring content validity. Except for problem-solving, the ISDS builds on current ASQ-3 development assessment tools' expertise by altering the structure of its related areas of communication, gross motor, fine motor, and personal-social. However, each domain of ISDS has a different number of items ranging from 3 to 5, whereas ASQ-3 has a set of six items, although parents score the presence of each skill in a comparable way as “Yes,” “Sometimes,” or “Not Yet” with point values of 10, 5, or 0.

### Strengths and limitations

One notable strength of this study was the inclusion of samples of Nigerian children from newborn care and immunisation clinics in Ibadan. Except for a few (1.3 percent) mothers who voiced concern about their children's vision or behaviours that were not clinically significant, these children were supposedly healthy. The fact that we conducted a test-retest reliability study with a different sample population adds to the validity of the new ISDS. Regardless of the familiarity with the content and arousal level of the children, the ISDS charts can be administered by caregivers or health workers, and it is certain that the findings of the tests will be reliable.

## Conclusion

Our study resulted in a simple, valid, and effective screening tool for the early identification of children who are at risk of developmental delay and thus require further developmental evaluation and subsequent individualised intervention. Future work should include the wider population deployment of the tool using digital health approaches that could underpin policy making in the region.

## Data Availability

The raw data supporting the conclusions of this article will be made available by the authors, without undue reservation.
