# Supplementary material for: Development and validation of the Ibadan Simplified Developmental Screening chart
Source: Front Pediatr. 2023 Jan 11;10:1055997. doi: 10.3389/fped.2022.1055997 (PMC9930897; doi:10.3389/fped.2022.1055997)
Supplement: Supplementary file 1 [file Datasheet1.pdf]

IBADAN SIMPLIFIED DEVELOPMENTAL SCREENING (ISDS) CHART

NAME ____

DATE OF EVALUATION _______________ DATE OF BIRTH ______________ AGE ____________

GESTATIONAL AGE AT DELIVERY _________ (WEEKS) PRETERM/TERM DELIVERY __________

ANY PARENTAL CONCERNS ____

LIST : ____

NAME OF ATTENDING PHYSICIAN ____

NAME OF CONSULTANT ____

6 WEEKS

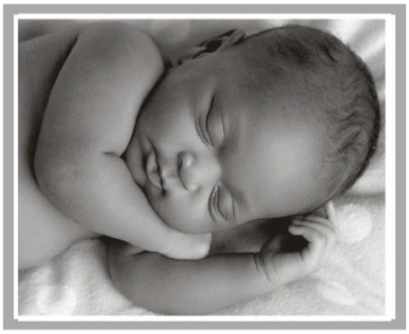

	Yes	*Somewhat*	*No*
**Vision and Fine motor**			
• Can baby get his fist to his mouth?	☐	☐	☐
• Does baby move both hands freely?	☐	☐	☐
• Does baby focus and follow on your face?	☐	☐	☐
• Does baby turn head and eyes to a light source?	☐	☐	☐
Vision and Fine Motor Total			☐
**Hearing, speech and language**			
• Does baby react to loud sound with a startle?	☐	☐	☐
• Does baby pay attention to speaker's mouth or eyes?	☐	☐	☐
• Does baby coo smile in response to face or voice?	☐	☐	☐
Hearing, speech and language Total			☐
**Social, emotional and behavioural**			
• Does baby look at you in the eye for a few seconds?	☐	☐	☐
• Does baby suck well?	☐	☐	☐
• Does baby smile responsively?	☐	☐	☐
Social, emotional and behavioural Total			☐
**Gross motor**			
• When baby is pulled from lying to sitting, does he drop head to the back - so you need to support his head?	☐	☐	☐
• Does baby lift his head up momentarily when lying tummy down?	☐	☐	☐
• When baby is held in standing position, does he lift one foot after another in stepping pattern?	☐	☐	☐
Gross motor Total			☐

Lagunju IA, Adeniyi Y, Orimadegun AE, and Fernandez-Reyes D (2022)

IBADAN SIMPLIFIED DEVELOPMENTAL SCREENING (ISDS) CHART

NAME____

DATE OF EVALUATION ________________ DATE OF BIRTH______________ AGE____________

GESTATIONAL AGE AT DELIVERY_________(WEEKS) PRETERM/TERM DELIVERY__________

ANY PARENTAL CONCERNS ____

LIST :____

NAME OF ATTENDING PHYSICIAN ____

NAME OF CONSULTANT ____

10 WEEKS

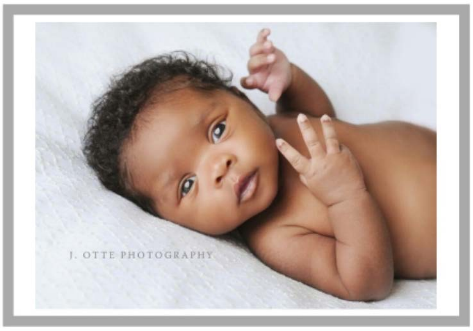

**Vision and Fine Motor**	*Yes*	*Somewhat*	*No*
• Does baby keep hands opened and relaxed most of the time?	☐	☐	☐
• When hand or object is moved close to his eyes,	☐	☐	☐
does he blink in defence?	☐	☐	☐
• Does child follow faces by moving eyes from side to side?	☐	☐	☐
Vision and Fine motor Total			☐

**Hearing, speech and language**			
• Does baby move, usually the upper limbs, to familiar voice?	☐	☐	☐
• Has baby started making single vowel sounds "ah""eh""uh"?	☐	☐	☐
• Does baby listen to voice and coos?	☐	☐	☐
• Does baby make sounds to caregiver's smile and voice?	☐	☐	☐
Hearing, speech and language total			☐

**Social, emotional and behavioural**			
• Does baby make cooing sounds? (soft, murmuring sound)	☐	☐	☐
• Does babyshow excitement by waving arms and legs?	☐	☐	☐
• Does baby give warm smiles and laughs?	☐	☐	☐
Social, emotional and behavioural			☐

**Gross motor**			
• When child is laid on his tummy, does he raise head from the floor?	☐	☐	☐
• Does the head lag when pulled to the sitting position?	☐	☐	☐
Gross motor total			☐

IBADAN SIMPLIFIED DEVELOPMENTAL SCREENING (ISDS) CHART

NAME____

DATE OF EVALUATION_____________ DATE OF BIRTH___________ AGE_________

GESTATIONAL AGE AT DELIVERY_______(WEEKS) PRETERM/TERM DELIVERY________

ANY PARENTAL CONCERNS____

LIST:____

NAME OF ATTENDING PHYSICIAN_______________________________________

NAME OF CONSULTANT____

14 WEEKS

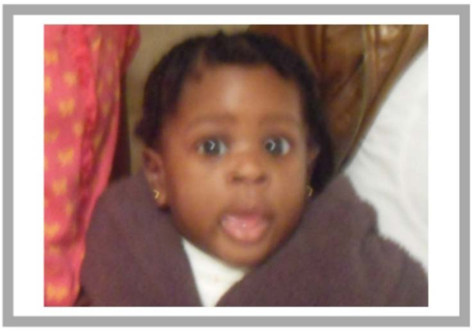

	Yes	*Somewhat*	*No*
**Vision and Fine Motor**			
• Does child reach out for toys?	☐	☐	☐
• Can child hold toys at least briefly?	☐	☐	☐
• Move her arms together and apart?	☐	☐	☐
Vision and Fine Motor total			☐
**Hearing, speech and language**			
• Does child turn head to search for sounds/listen to voice?	☐	☐	☐
• Does child recognise caregiver's voice?	☐	☐	☐
• Does child vocalize "ma" or "da"?	☐	☐	☐
Hearing, speech and language total			☐
**Social, emotional and behavioural**			
• Does child smile both spontaneously and back at people?	☐	☐	☐
• Does child cry when upset and seek comfort?	☐	☐	☐
• Does child smile at herself in the mirror?	☐	☐	☐
Social, emotional and behavioural total			☐
**Gross motor**			
• When lying on his back, can child roll from back to side?	☐	☐	☐
• Does child hold head steady when held in sitting position?	☐	☐	☐
• When held in sitting position, can child reach for toys around him/her?	☐	☐	☐
Gross motor total			☐

Lagunju IA, Adeniyi Y, Orimadegun AE, Fernandez-Reyes D (2022)

IBADAN SIMPLIFIED DEVELOPMENTAL SCREENING (ISDS) CHART

NAME ____

DATE OF EVALUATION ________________ DATE OF BIRTH ______________ AGE ___________

GESTATIONAL AGE AT DELIVERY ________(WEEKS) PRETERM/TERM DELIVERY __________

ANY PARENTAL CONCERNS ____

LIST : ____

NAME OF ATTENDING PHYSICIAN ____

NAME OF CONSULTANT ____

6 MONTHS

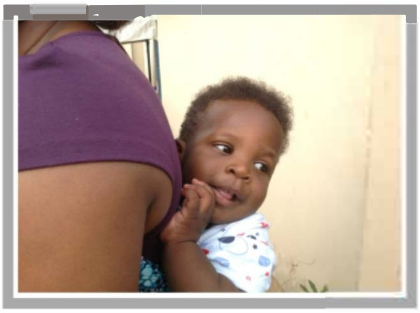

	Yes	Somewhat	No
**Fine motor and Vision**			
• Does baby follows objects with his eyes in different directions?			
• Does babyreach out for objects?			
• Can baby pass objects from one hand to the other hand?			
• When lying on the back, can baby touch knees and feet with hands?			
Fine motor and Vision total			
**Hearing, Speech and Language**			
• Can baby manipulate a rattle?			
• Can baby differentiate between familiar and strange voices?			
• Does baby bang objects to elicit sound?			
• Does baby turn eyes/head to source of sound?			
Hearing, speech and language total			
**Social, emotional and behavioural**			
• Does baby babble?			
• Does baby respond to own name?			
• Does baby cry or show displeasure at the loss of a toy?			
Social, emotional and behavioural total			
**Gross motor**			
• When lying on his tummy, can baby raise head and upper part of the body?			
• When lying on his back, can he/she roll to the front?			
• Can baby sit with little or no support?			
Gross motor total			

IBADAN SIMPLIFIED DEVELOPMENTAL SCREENING (ISDS) CHART

NAME ____

DATE OF EVALUATION ______________ DATE OF BIRTH ____________ AGE __________

GESTATIONAL AGE AT DELIVERY ________(WEEKS) PRETERM/TERM DELIVERY __________

ANY PARENTAL CONCERNS ____

LIST: ____

NAME OF ATTENDING PHYSICIAN ____

NAME OF CONSULTANT ____

9 MONTHS

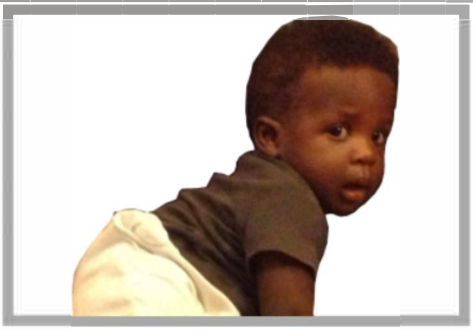

	Yes	*Somewhat*	*No*
**Fine motor and Vision**			
· Can baby pick up toys with only one hand?	☐	☐	☐
· Does baby reach out, grab, and put objects in his mouth?	☐	☐	☐
· Can baby put objects into containers (with large openings) and take them out again	☐	☐	☐
Fine motor and vision total			☐
**Hearing, Speech and Language**			
· Does baby turns eye/head/body to source of sound?	☐	☐	☐
· Does baby respond to name?	☐	☐	☐
· Does baby attempt to sing along with a familiar song/babble?	☐	☐	☐
Hearing, speech and language total			☐
**Social, emotional and behavioural**			
· Does baby cry when strangers approach?	☐	☐	☐
· When baby is upset, does he show ways of comforting self like sucking thumb, holding on to a favourite toy, etc?	☐	☐	☐
· Does baby show excitement at the sight of other babies?	☐	☐	☐
Social, emotional and behavioural total			☐
**Gross motor**			
· Does baby get into crawling position?	☐	☐	☐
· Does baby reach out for toys at a distance when in sitting position?	☐	☐	☐
· Is baby able to sit without support and without falling over?	☐	☐	☐
Gross motor total			☐

IBADAN SIMPLIFIED DEVELOPMENTAL SCREENING (ISDS) CHART

NAME____

DATE OF EVALUATION________________ DATE OF BIRTH______________ AGE___________

GESTATIONAL AGE AT DELIVERY_________(WEEKS) PRETERM/TERM DELIVERY__________

ANY PARENTAL CONCERNS____

LIST:____

NAME OF ATTENDING PHYSICIAN____

NAME OF CONSULTANT____

12 MONTHS

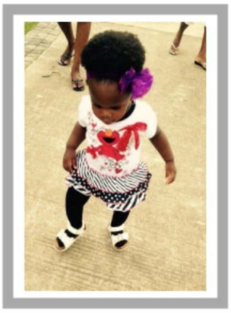

	Yes	Somewhat	No
**Fine motor and Vision**			
· Can baby pick medium-sized bead/button with the thumb and another finger?	☐	☐	☐
· Can baby give toy to caregiver when asked?	☐	☐	☐
· Can baby hold/attempt to hold hisown bottle/cup?	☐	☐	☐
· Is baby able to build a block of 2 cubes?	☐	☐	☐
Fine motor and vision total			☐
**Hearing, Speech and Language**			
· Does baby say “mama” and “dada”?	☐	☐	☐
· Does baby try to imitate words?	☐	☐	☐
· Can baby identify two body parts on self?	☐	☐	☐
Hearing, speech and language total			☐
**Social, emotional and behavioural**			
· Can baby wave bye bye?	☐	☐	☐
· Does baby use simple gesture like shaking head to mean "no"?	☐	☐	☐
· Does baby cry when care giver leaves?	☐	☐	☐
Social, emotional and behavioural total			☐
**Gross motor**			
· Can baby cruise around furniture?	☐	☐	☐
· Can baby lower self to pick toys while holding on to furniture?	☐	☐	☐
· Can baby pull himself to a standing position while sitting?	☐	☐	☐
Gross motor total			☐
