# Supplementary material for: Development and validation of the Ibadan Simplified Developmental Screening chart
Source: Front Pediatr. 2023 Jan 11;10:1055997. doi: 10.3389/fped.2022.1055997 (PMC9930897; doi:10.3389/fped.2022.1055997)
Supplement: Supplementary file 2 [file Datasheet2.pdf]

IBADAN SIMPLIFIED DEVELOPMENTAL SCREENING (ISDS) CHART SCORING GUIDE

AGE – 6 WEEKS

1. EVALUATE EVERY SINGLE ACTIVITY
2. ALLOCATE THE SCORE FOR EACH ACTIVITY IN EACH DOMAIN OF DEVELOPMENT
3. YES = 5, SOMEWHAT = 2, NO = 0
4. ADD UP THE SCORES FOR ALL THE ACTIVITIES IN EACH DOMAIN TO ARRIVE AT THE TOTAL DOMAIN SCORE
5. PUT SCORE IN THE CORRESPONDING BOX TO DETERMINE PERFORMANCE
6. IF CHILD'S SCORE FALLS IN THE RED BOX, REFER FOR FURTHER EVALUATION
7. IF CHILD'S SCORE FALLS IN THE PLAIN BOX, CHILD IS DOING WELL AND DOES NOT REQUIRE FURTHER EVALUATION AT THE TIME

**Domain of Development**	**0**	**1**	**2**	**3**	**4**	**5**	**6**	**7**	**8**	**9**	**10**	**11**	**12**	**13**	**14**	**15**	**16**	**17**	**18**	**19**	**20**	**21**	**22**	**23**	**24**	**25**
Vision & Fine motor																										
Hearing speech and Language																										
Social, emotional																										
Gross motor																										

Lagunju IA, Adeniyi Y, Orimadegun AE, Fernandez-Reyes D (2022)

IBADAN SIMPLIFIED DEVELOPMENTAL SCREENING (ISDS) CHART SCORING GUIDE

AGE – 10 WEEKS

1. EVALUATE EVERY SINGLE ACTIVITY
2. ALLOCATE THE SCORE FOR EACH ACTIVITY IN EACH DOMAIN OF DEVELOPMENT
3. YES =5, SOMEWHAT = 2, NO = 0
4. ADD UP THE SCORES FOR ALL THE ACTIVITIES IN EACH DOMAIN TO ARRIVE AT THE TOTAL DOMAIN SCORE
5. PUT SCORE IN THE CORRESPONDING BOX TO DETERMINE PERFORMANCE
6. IF CHILD'S SCORE FALLS IN THE RED BOX, REFER FOR FURTHER EVALUATION
7. IF CHILD'S SCORE FALLS IN THE PLAIN BOX, CHILD IS DOING WELL AND DOES NOT REQUIRE FURTHER EVALUATION AT THE TIME

**Domain of Development**	**0**	**1**	**2**	**3**	**4**	**5**	**6**	**7**	**8**	**9**	**10**	**11**	**12**	**13**	**14**	**15**	**16**	**17**	**18**	**19**	**20**	**21**	**22**	**23**	**24**	**25**
Vision & Fine motor																										
Hearing speech and Language																										
Social, emotional, behavioural																										
Gross motor																										

Lagunju IA, Adeniyi Y, Orimadegun AE, Fernandez-Reyes D (2022)

IBADAN SIMPLIFIED DEVELOPMENTAL SCREENING (ISDS) CHART SCORING GUIDE

AGE – 14 WEEKS

1. EVALUATE EVERY SINGLE ACTIVITY
2. ALLOCATE THE SCORE FOR EACH ACTIVITY IN EACH DOMAIN OF DEVELOPMENT
3. YES =5, SOMEWHAT = 2, NO = 0
4. ADD UP THE SCORES FOR ALL THE ACTIVITIES IN EACH DOMAIN TO ARRIVE AT THE TOTAL DOMAIN SCORE
5. PUT SCORE IN THE CORRESPONDING BOX TO DETERMINE PERFORMANCE
6. IF CHILD'S SCORE FALLS IN THE RED BOX, REFER FOR FURTHER EVALUATION
7. IF CHILD'S SCORE FALLS IN THE PLAIN BOX, CHILD IS DOING WELL AND DOES NOT REQUIRE FURTHER EVALUATION AT THE TIME

**Domain of Development**	**0**	**1**	**2**	**3**	**4**	**5**	**6**	**7**	**8**	**9**	**10**	**11**	**12**	**13**	**14**	**15**	**16**	**17**	**18**	**19**	**20**	**21**	**22**	**23**	**24**	**25**
Vision & Fine motor																										
Hearing speech and Language																										
Social, emotional, behavioural																										
Gross motor																										

Lagunju IA, Adeniyi Y, Orimadegun AE, Fernandez-Reyes D (2022)

IBADAN SIMPLIFIED DEVELOPMENTAL SCREENING (ISDS) CHART SCORING GUIDE

AGE – 6 MONTHS

1. EVALUATE EVERY SINGLE ACTIVITY
2. ALLOCATE THE SCORE FOR EACH ACTIVITY IN EACH DOMAIN OF DEVELOPMENT
3. YES =5, SOMEWHAT = 2, NO = 0
4. ADD UP THE SCORES FOR ALL THE ACTIVITIES IN EACH DOMAIN TO ARRIVE AT THE TOTAL DOMAIN SCORE
5. PUT SCORE IN THE CORRESPONDING BOX TO DETERMINE PERFORMANCE
6. IF CHILD'S SCORE FALLS IN THE RED BOX, REFER FOR FURTHER EVALUATION
7. IF CHILD'S SCORE FALLS IN THE PLAIN BOX, CHILD IS DOING WELL AND DOES NOT REQUIRE FURTHER EVALUATION AT THE TIME

**Domain of Development**	**0**	**1**	**2**	**3**	**4**	**5**	**6**	**7**	**8**	**9**	**10**	**11**	**12**	**13**	**14**	**15**	**16**	**17**	**18**	**19**	**20**	**21**	**22**	**23**	**24**	**25**
Vision & Fine motor																										
Hearing speech and Language																										
Social, emotional, behavioural																										
Gross motor																										

Lagunju IA, Adeniyi Y, Orimadegun AE, Fernandez-Reyes D (2022)

IBADAN SIMPLIFIED DEVELOPMENTAL SCREENING (ISDS) CHART SCORING GUIDE

AGE – 9 MONTHS

1. EVALUATE EVERY SINGLE ACTIVITY
2. ALLOCATE THE SCORE FOR EACH ACTIVITY IN EACH DOMAIN OF DEVELOPMENT
3. YES =5, SOMEWHAT = 2, NO = 0
4. ADD UP THE SCORES FOR ALL THE ACTIVITIES IN EACH DOMAIN TO ARRIVE AT THE TOTAL DOMAIN SCORE
5. PUT SCORE IN THE CORRESPONDING BOX TO DETERMINE PERFORMANCE
6. IF CHILD'S SCORE FALLS IN THE RED BOX, REFER FOR FURTHER EVALUATION
7. IF CHILD'S SCORE FALLS IN THE PLAIN BOX, CHILD IS DOING WELL AND DOES NOT REQUIRE FURTHER EVALUATION AT THE TIME

**Domain of Development**	**0**	**1**	**2**	**3**	**4**	**5**	**6**	**7**	**8**	**9**	**10**	**11**	**12**	**13**	**14**	**15**	**16**	**17**	**18**	**19**	**20**	**21**	**22**	**23**	**24**	**25**
Vision & Fine motor																										
Hearing speech and Language																										
Social, emotional, behavioural																										
Gross motor																										

Lagunju IA, Adeniyi Y, Orimadegun AE, Fernandez-Reyes D (2022)

IBADAN SIMPLIFIED DEVELOPMENTAL SCREENING (ISDS) CHART SCORING GUIDE

AGE – 12 MONTHS

1. EVALUATE EVERY SINGLE ACTIVITY
2. ALLOCATE THE SCORE FOR EACH ACTIVITY IN EACH DOMAIN OF DEVELOPMENT
3. YES =5, SOMEWHAT = 2, NO = 0
4. ADD UP THE SCORES FOR ALL THE ACTIVITIES IN EACH DOMAIN TO ARRIVE AT THE TOTAL DOMAIN SCORE
5. PUT SCORE IN THE CORRESPONDING BOX TO DETERMINE PERFORMANCE
6. IF CHILD'S SCORE FALLS IN THE RED BOX, REFER FOR FURTHER EVALUATION
7. IF CHILD'S SCORE FALLS IN THE PLAIN BOX, CHILD IS DOING WELL AND DOES NOT REQUIRE FURTHER EVALUATION AT THE TIME

**Domain of Development**	**0**	**1**	**2**	**3**	**4**	**5**	**6**	**7**	**8**	**9**	**10**	**11**	**12**	**13**	**14**	**15**	**16**	**17**	**18**	**19**	**20**	**21**	**22**	**23**	**24**	**25**
Vision & Fine motor																										
Hearing speech and Language																										
Social, emotional, behavioural																										
Gross motor																										

Lagunju IA, Adeniyi Y, Orimadegun AE, Fernandez-Reyes D (2022)
